# Ventricular septal defect associated with aortic regurgitation and ascending aortic aneurysm: a case report

**DOI:** 10.1186/s13256-023-04167-7

**Published:** 2023-10-26

**Authors:** Edmond Haliti, Besim Bytyçi, Michael Y. Henein, Gani Bajraktari, Ibadete Bytyçi

**Affiliations:** 1 Clinic of Cardiology, University Clinical Centre of Kosova, Rrethi i Spitalit, pn., 10000 Prishtina, Republic of Kosovo; 2 Clinic of Rheumatology, University Clinical Centre of Kosova, Prishtina, Republic of Kosovo; 3 Department of Public Health and Clinical Medicine, Umeå University, Umeå, Sweden

**Keywords:** Inter-ventricular septal defect, Aortic regurgitation, Venturi effect, Ascending aortic aneurysm

## Abstract

**Introduction:**

Ventricular septal defect (VSD) is one of the most common congenital cardiac anomalies. Patients with perimembranous VSD may have aortic regurgitation (AR) secondary to prolapse of the aortic cusp.

**Case presentation:**

We present a case of 23-year-old White man with VSD, AR and ascending aortic aneurysm. The patient presented to outpatient clinic with weakness and gradual worsening shortness of breath for the past 5 years. Clinical examination revealed regular heart rhythm and loud continuous systolic-diastolic murmur (Lewin’s grade 6/6), heard all over the precordium, associated with a palpable thrill. The ECG showed right axis deviation, fractionated QRS in V1 and signs of biventricular hypertrophy. The chest X-ray showed cardiomegaly. Transthoracic and transesophageal echocardiograms showed a perimembranous VSD with moderate restrictive shunt (Qp/Qs = 1.6), aortic regurgitation (AR), and ascending aortic aneurysm. Other clinical and laboratory findings were within normal limits.

**Conclusions:**

Perimembranous VSD, may be associated with aortic regurgitation and ascending aortic aneurysm as secondary phenomenon if it is not early diagnosed and successfully treated.

**Supplementary Information:**

The online version contains supplementary material available at 10.1186/s13256-023-04167-7.

## Introduction

Ventricular septal defect (VSD) is one of the most common congenital cardiac anomalies both in children and adults [1]. VSD in childhood occurs in 50% of all congenital heart disease, and in adults, despite being rare. It is the second most prevalent congenital heart disease, after bicuspid aortic valve [2]. The cause of VSD and other congenital heart diseases is not fully understood, but it is now widely accepted that they are genetically mediated and potentially influenced by exogenic factors during fetal development, leading to disruption of the delicate and complex process of normal heart and great vessel morphogenesis.

VSDs can be classified according to their location, size, hemodynamic impact and accompaniment with other anomalies [3]. Because of the close anatomical relation of membranous interventricular septum (IVS) and the semilunar aortic valve, aortic regurgitation (AR) is seen in patients with perimembranous subtypes of VSD, caused by the secondary prolapse as a result of Venturi effect [4]. Delay in diagnosis and treatment of perimembranous VSD can cause physiological stress on the aorta and valve that can lead to AR and aneurysm.

## Case presentation

We present a 23-years-old White man with VSD and AR, associated with ascending aortic aneurysm. The patient was admitted because of generalized. weakness and worsening exertional breathlessness, over the previous five years, with significant deterioration in last year. His personal and family history denied significant cardiovascular risk factors.

Clinical examination revealed regular heart rhythm, loud continuous systolic-diastolic murmur (Lewin’s grade 6/6) heard over the whole precordium with *punctum maximum* on the left sternal border, associated with a palpatory thrill.

**Fig. 1:**
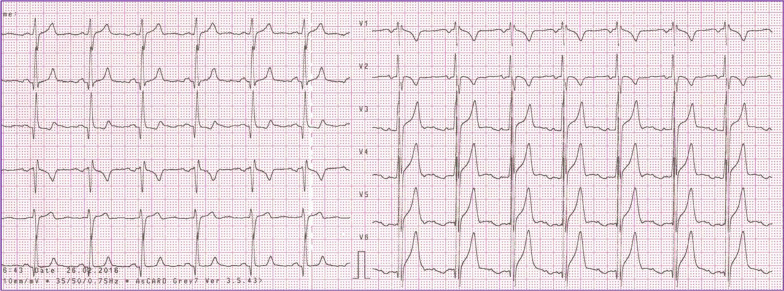
Biventricular hypertrophy on electrocardiogram of the patient

A 12 lead ECG showed slight right axis deviation, fractionated QRS in V1 and electric criteria for biventricular hypertrophy (Fig. 1).

**Fig. 2:**
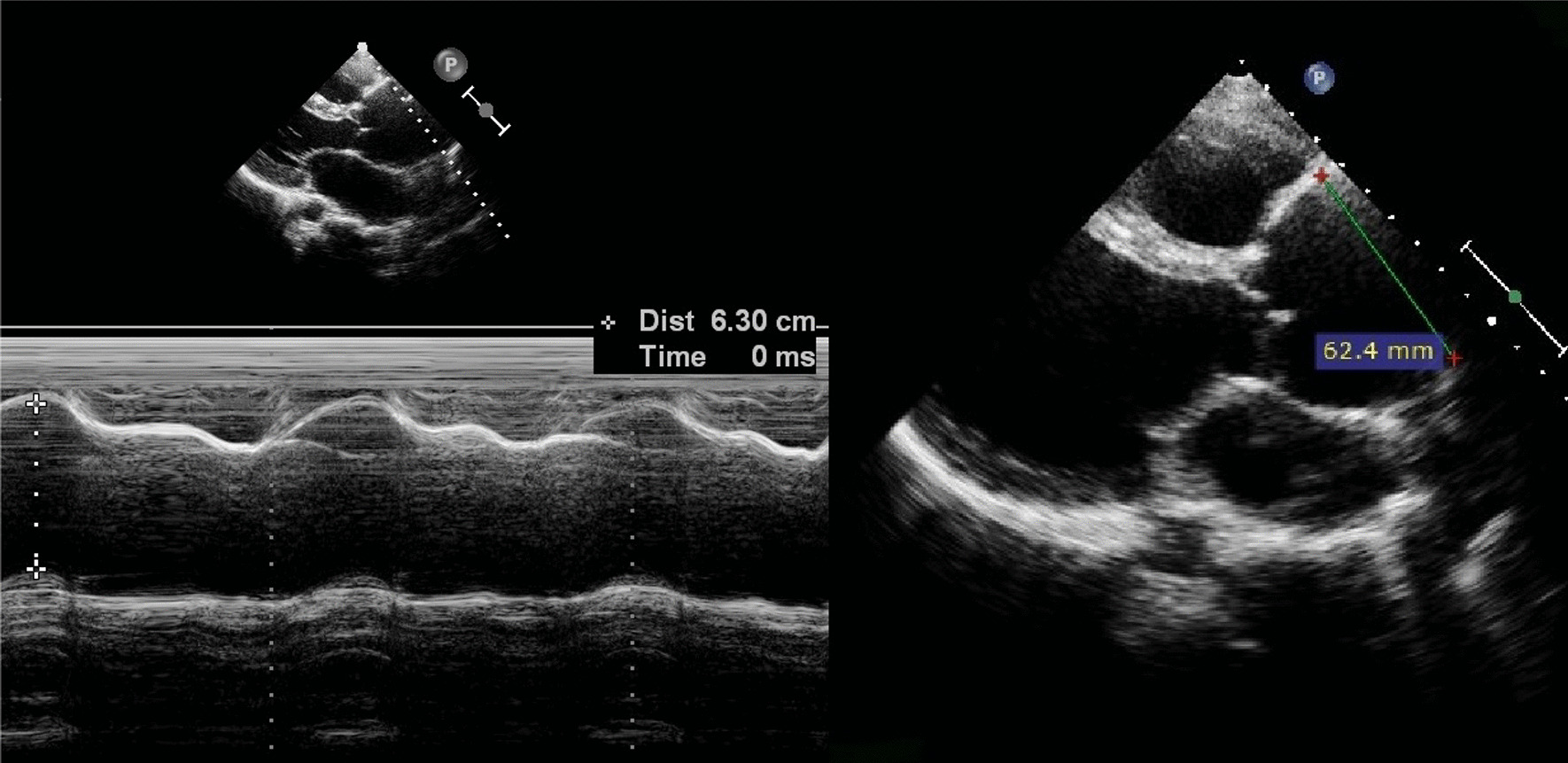
Echocardiogram/M-mode and 2D measurements of ascendent aorta

**Fig. 3:**
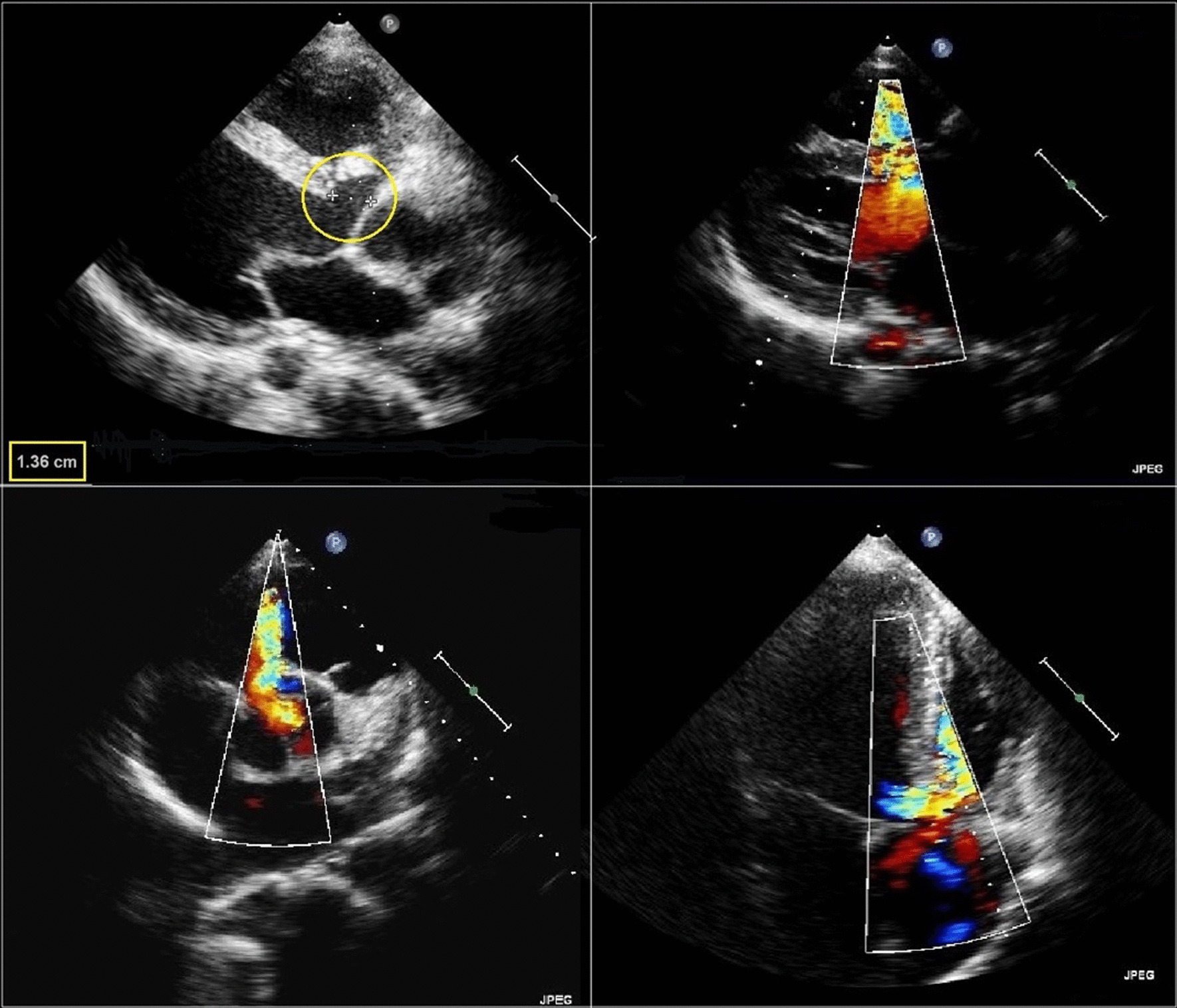
Color-Doppler imaging of ventricular septal defect in the parasternal and apical wiew

**Fig. 4:**
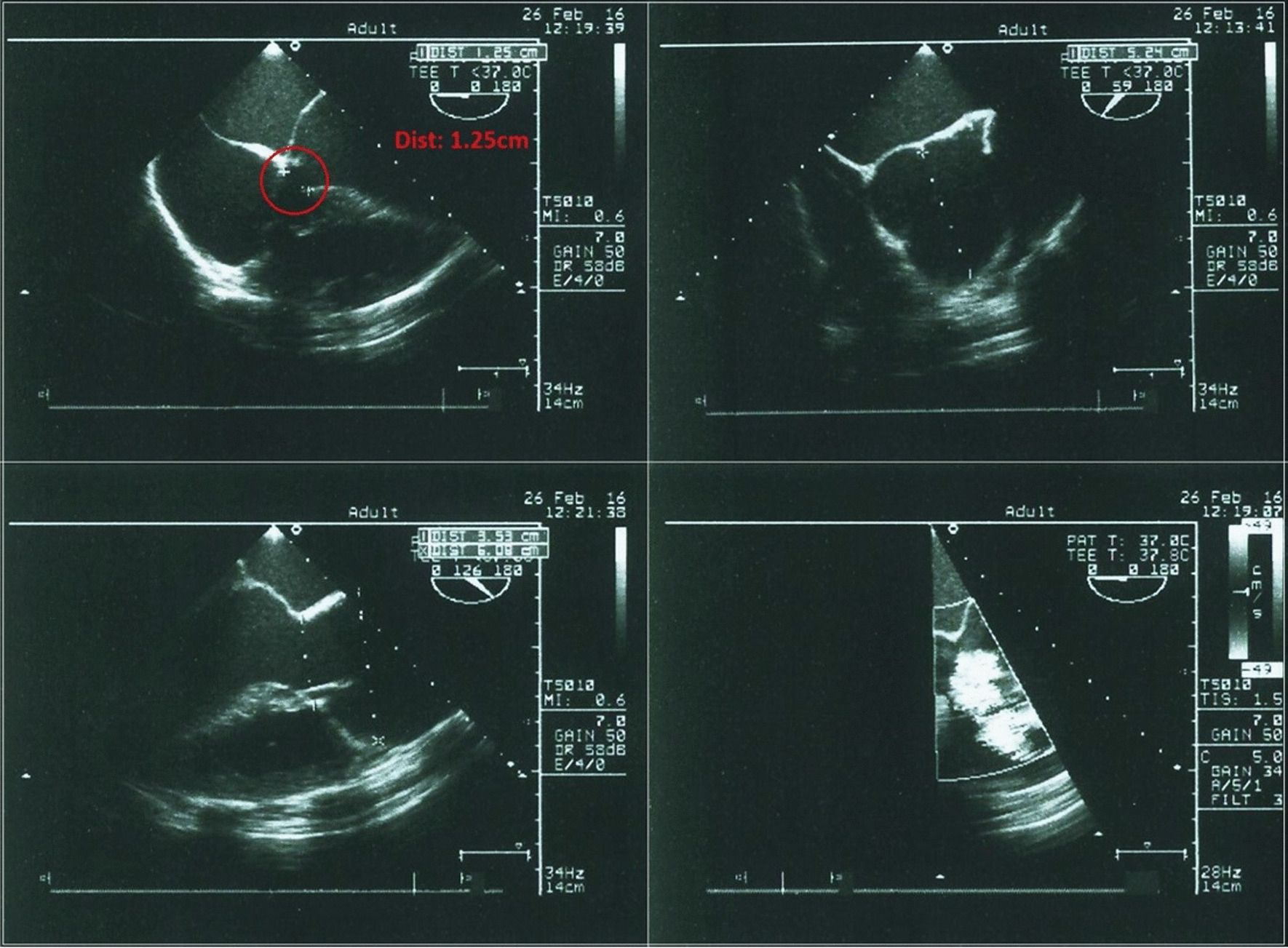
Transesophageal echocardiography views

Chest X- ray showed enlarged cardiac silhouette suggestive of left ventricular hypertrophy. Other clinical and laboratory findings were within normal range. A transthoracic echocardiogram demonstrated a perimembranous VSD, with moderate restrictive shunt (Qp/Qs = 1.6), AR and ascending aortic aneurysm (Fig. 2, Additional file 1: Video S1). Similar findings were confirmed by transesophageal echocardiogram (Figs. 3, 4).

## Discussion

Congenital VSD may be found as an isolated lesion or in combination with other cardiac anomalies [5]. As for all congenital heart disease, there are some genetical disorders responsible for the formation of VSDs during the intrauterine fetal development, which can lead to a disruption of a very sensitive and complex process of normal morphogenesis of heart and great vessels. These genetic disorders may also be related to teratogenic exogenous factors [6].

The interventricular septum (IVS) is quite a voluminous muscular structure that separates the two ventricles, despite the distinguishable basal membranous septum which is in close proximity to the atrio-ventricular and semilunar aortic valves. The muscular part of the IVS can be divided into inlet, trabecular and infundibular components [2, 5, 7].

Because of the close anatomical relation of the membranous IVS and the aortic valve, aortic regurgitation (AR) is seen in patients with perimembranous VSDs, being caused by secondary prolapse of one of the aortic valve cusps as a result of the Venturi effect [3, 5]. The natural history of these defects is a progressive deterioration of the semilunar cusp involved, resulting in worsening AR [8]. The absence of a muscular septum below the adjacent aortic valve cusp results in unopposed downward force on the cusp during diastole, hence cusp prolapse and AR. Yet larger VSDs, which are bordered by a correspondingly larger portion of the aortic annulus, rarely develop aortic valve prolapse or AR. This suggests that the lack of ventricular septal support alone is an inadequate explanation. If a lack of support were a principal cause of AR then it would follow that larger VSDs would, more commonly, result in AR. But according to the available literature, the opposite is true, indicating the predominance of the Venturi effect in the pathogenesis. Abnormal commissural structures have been observed in some cases of AR associated with a VSD [7].

Literature has disclosed the importance of an early diagnosis, follow-up and appropriate treatment of this condition as indicated in a certain stage of disease. Although some asymptomatic VSD are set for a very careful follow-up throughout childhood because, they were considered too small to warrant surgical intervention, there are some early evidence that the heart may be hyperkinetic, slightly overloaded, but `yet no distinct abnormality could be displayed by conventional diagnostic investigations [8]. Many adult patients with congenital VSD also develop ascending aortic dilation, but only few report the clinical features and surgical management of these patients [8].

We report a case of a 23 years man, with very lately diagnosed VSD, associated with AR and ascending aortic aneurysm. The patient had experienced generalized weakness and exertional breathlessness during the previous five years, with a marked decline in his symptoms in the last year. VSDs that result in congestive heart failure (HF) are routinely closed in infancy, whereas those associated with AR are nearly always in adults, as in our patient. The lack of early diagnosis and appropriate treatment worsens prognosis. VSD closure is indicated in cases of heart failure, large shunt, and elevated pulmonary artery pressure caused by hemodynamic changes, regardless of AR presence and severity. However, the development of AR may occur in the absence of congestive heart failure [9]. In the absence of a hemodynamically significant VSD, the indications for surgical repair become more complicated. Closure of the VSD, with or without aortic valve repair, is indicated for both perimembranous and subarterial VSDs when more than trivial AR is identified because of the progressive nature of AR. For patients with a subarterial VSD and AVP, VSD closure is indicated because of the high likelihood of progression of AVP and development of AR. Subarterial VSDs, larger than 5 mm should be closed regardless of the presence of aortic cusp prolapse, in order to prevent the development of AR. Furthermore, there is only a minimal chance for spontaneous closure of a subarterial VSD [10].

Types of surgical technique for VSD closure are few. In patients with doubly committed supracristal defect, in which the lack of support of the semilunar cusp seems to be the basic mechanism underlying the prolapse, simple closure of the defect with a patch should solve the problem. This technique would be ideal for cases with a small muscular ridge and conserved aortic ring. Highly evolved cases with a large prolapse require the placement of a double patch, one on the defect and the other on the aneurysm [11]. Owing to the anatomic relationship between the VSD and aortic structure, there might be some potential reoperations of aortic disease after VSD repair. So, it is extremely vital to address aortic disorder in patients with congenital VSD, especially when aortic cusps are involved [12].

## Conclusion

Perimembraneous VSD, may be associated with aortic regurgitation and ascending aortic aneurysm as a secondary phenomenon, in case that it is not early diagnosed and successfully treated. Early diagnosis and management of these patients should prevent disease worsening and hemodynamic deterioration.

### Supplementary Information

**Additional file 1:** Coexistence of ventricular septal defect, aortic regurgitation, and ascending aortic aneurysm; or Concurrent ventricular septal defect, aortic regurgitation, and ascending aortic aneurysm.

## Data Availability

Availability of supporting data.
